# 12p deletion spectrum syndrome: a new case report reinforces the evidence regarding the potential relationship to autism spectrum disorder and related developmental impairments

**DOI:** 10.1186/s13039-016-0278-0

**Published:** 2016-10-04

**Authors:** Marcio Leyser, Bruno Leonardo Dias, Ana Luiza Coelho, Marcio Vasconcelos, Osvaldo J. M. Nascimento

**Affiliations:** 1 The SARAH Network of Neurorehabilitation Hospitals-SARAH International Center for Neurorehabilitation and Neuroscience, Avenida Abelardo Bueno, n° 1500, ZIP:22775-040 Rio de Janeiro, RJ Brazil; 2 Antonio Pedro University Hospital/Federal Fluminense University, Niterói, Brazil

**Keywords:** Autism spectrum disorder, 12p13.2 microdeletion, 12p microdeletion spectrum, Developmental delay

## Abstract

**Background:**

Autism Spectrum Disorders (ASD) now encompass a broad heterogeneous group of people who present in the early developmental years with a wide range of social and communication deficits, which are typically also associated with complex repetitive behaviors and circumscribed interests.

The target goal is to heighten readers’ perception into the trend to personalize the distinct autistic and related developmental conditions encompassing the 12p region.

**Case Presentation:**

This is a case-report of a 4-year-old male who presented the core signs of ASD, which were thought to be related to a rare 12p13.2 deletion.  We further reviewed the literature in order to outline the related developmental conditions in the 12p region.

Aside from this patient reported here, we found an additional number of 43 cases described in the medical literature since 1974, that have been related to deletions in the 12p region. However, to the best of our knowledge, none of the previous had been specifically linked to the 12p13.2 band.

**Conclusions:**

The 12p deletion spectrum is rarely described as part of the selective genotypes thought to be related to ASD. Even inside of a small piece of the puzzle, there might be ample variation in the behavioral and clinical phenotypes of children and adults presenting with this particular genetic profile.

In that regard, the particular 12p13.2 distal deletion presentation is one of the possible genotypes encompassed by the “12p deletion spectrum syndrome”, that might be potentially connected to the diagnosis of ASD and related developmental disorders.

## Background

Autism Spectrum Disorders (ASD) now encompass a broad heterogeneous group of people who present in the early developmental years with a wide range of social and communication deficits, which are typically also associated with complex repetitive behaviors and circumscribed interests [1].

With the advent of genomic technologies, studies have recently demonstrated that there is a strong heritability in ASD, as well as a positive interplay among genetic and environmental factors in the etiology of social deficits and unusual behaviors [2, 3]. Moreover, a high (60 to 90 %) concordance rate in monozygotic twins for ASD has been already determined [4].

The accumulation of these data overtime made a distinction between idiopathic (Non-syndromic) ASD and syndromic ASD possible. From Mendelian inheritance to *de novo* SNV and CNV *point* mutations [2] many genes are now believed to be implicated in the role of a neuronal molecular level activity [5]. These genes have also accounted for the neurobiological changes in part of the brain that affects social cognition, sensory perception and executive function [1, 6].

The trend toward linking autistic phenotypic behaviors to different genotypes is legitimate, but can be very unreliable due to changes in behavioral phenotype and developmental trajectories over time as individuals with ASD grow older [7].

The rarely reported terminal 12p deletion zone spectrum is a group of characteristic genotypes thought to be associated with autistic core features among other developmental, psychiatric, cancer predisposition and clinical phenotypic presentations [8–11].

The first report on the so-called “12p deletion spectrum” was published in 1975 by Magnelli and Therman [12, 13].

For clinical understanding purpose, the 12p regions can be divided into four group types according to the site of the interstitial deletion: 12p1-11; distantly extending deletions 12p11-13, 12p13 band, and the distal zone of 12p [14].

In this report, we present a new case of a 4-year-old with ASD and a 12p13.2 deletion, and further discuss the relationship of the condition to the phenotypic spectrum of the 12p region, by illustrating examples taken from the literature.

The target goal here is to heighten readers’ perception into the trend to personalize the distinct autistic and related developmental conditions encompassing the 12p region.

## Methods

### Patient recruitment

We recruited a 4-year-old boy diagnosed with developmental arrest and ASD due to an underlying 12p deletion for this report. Permission by the child’s mother has been granted through an informed consent, which has been approved by the SARAH Network Institutional Research Ethical Board under number 49915515.1.0000.0022.

A description of the case, as well as the results of the patient’s diagnostic tests has been undertaken below.

### Review of the literature

We searched PubMed for all existent articles related to interstitial and terminal deletions in the 12p region. The terms 12p and autism were used for this search. Only articles written in English, French and German were considered for a review. Fifty-two relevant papers were further completely appraised.

## Case Presentation

**Fig. 1:**
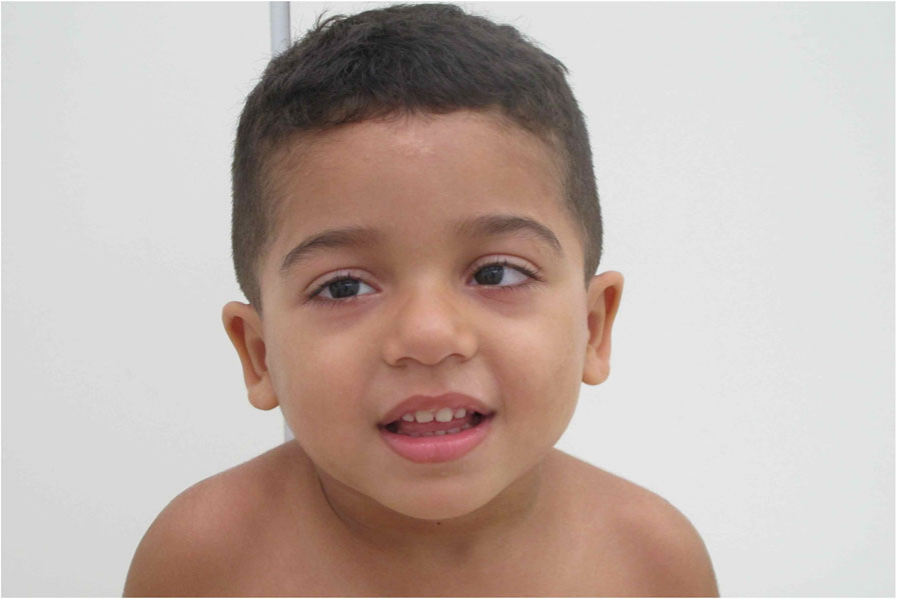
Anterior view of patient´s face and body

**Fig. 2:**
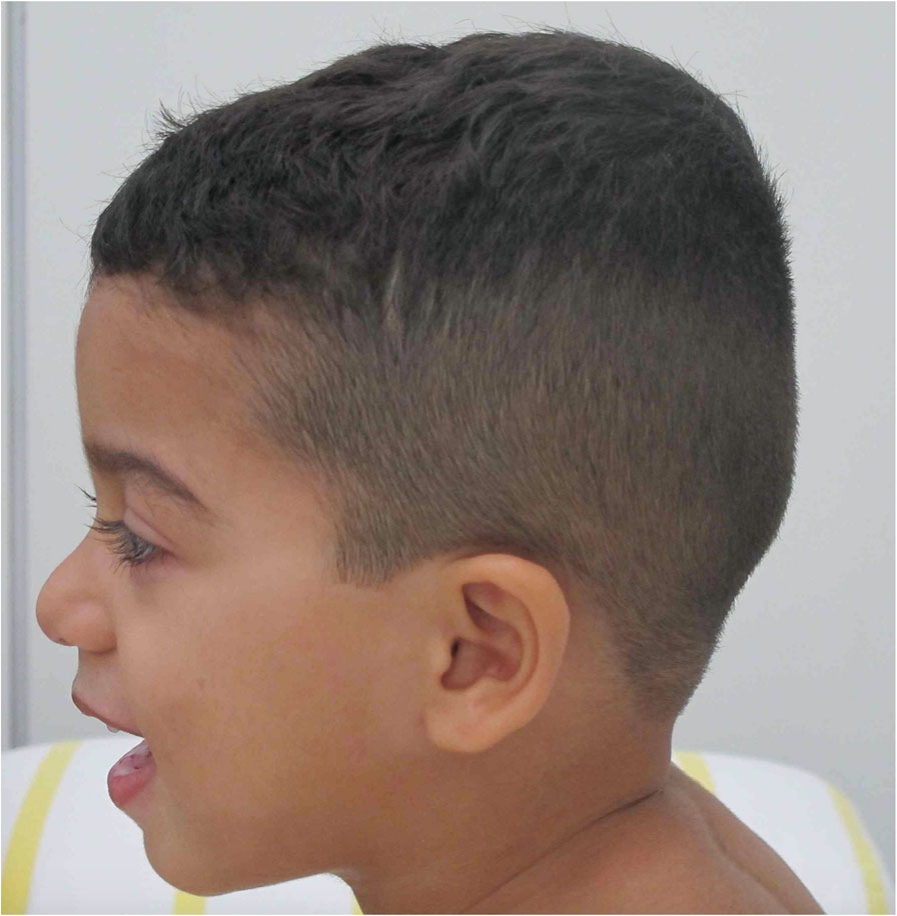
Lateral view of patient´s face and body

**Fig. 3:**
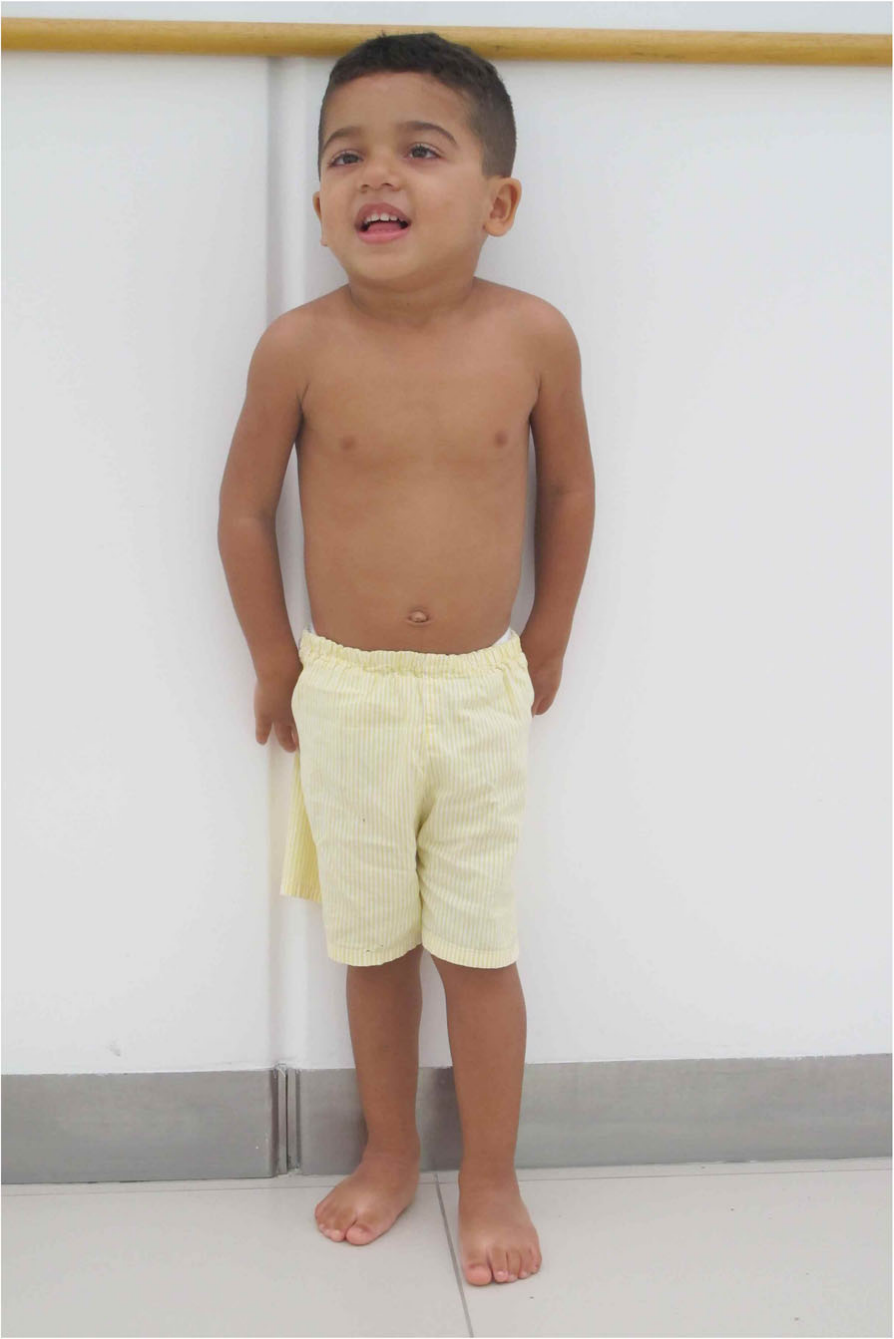
Anterior view of patient´s face and body

A 4-year-old male proband (Figs. 1, 2, 3) presented with global developmental delay noticed by his family when he was around 11 months of age. The child was born at term from a vaginal delivery and an uneventful pregnancy. His parents were non-consanguineous and healthy. The mother disclosed she had consumed alcohol for social purposes before being aware she was pregnant. She reports having drunk small beverages of beer during the week, as well as vodka throughout the weekends, until the third month of gestational period. The exact daily amount of alcohol consumption is unknown. Despite the fact the mother had a small amount of vaginal bleeding at 42 weeks of gestation, the child’s Apgar scores were 8 in the first and fifth minutes. The patient developed a mild, asymptomatic, hypoglycemia due to suction difficulties, but he was discharged from the hospital at day three of life and had been able to be breastfed until 3 months of age only.

The child’s social and language skills were below the rest of his developmental domains. He attained independent walking by 23 months of age. Nevertheless, his verbal and non-verbal communication capabilities were so weak that, at that age, it was clearly observed that the quality of his eye contact, as well as social interactions, were in the autistic spectrum range. Moreover, significant motor and vocal stereotypic behaviors alongside difficulties in functional play and imitation had also ensued. The child’s growth curves for weight (centile 15–25), height (centile 15–25) and head circumference (centile 75) have been steadily unchanged over the course of his growth. No facial or corporeal dysmorphic features have been detected, that could be specific for FAS, or any specific genetic syndrome. In addition, there were no reports of clinical seizures in this patient. His physical and neurological examinations were unremarkable, except by the fact that he has developed a refraction error visual impairment. He was seen by an ophthalmologist who prescribed lenses accordingly.

**Fig. 4:**
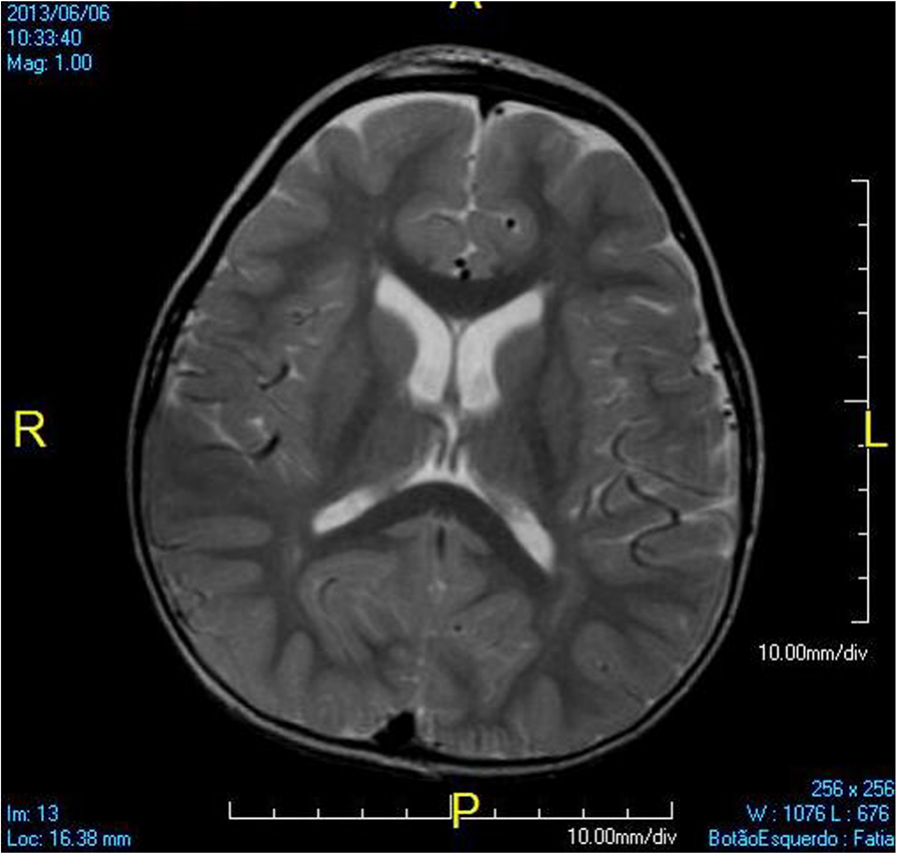
Brain MRI - Increased signal in T2-weighted images in the withe matter territories adjacent to the lateral ventricles bodies and subcortical zone

**Fig. 5:**
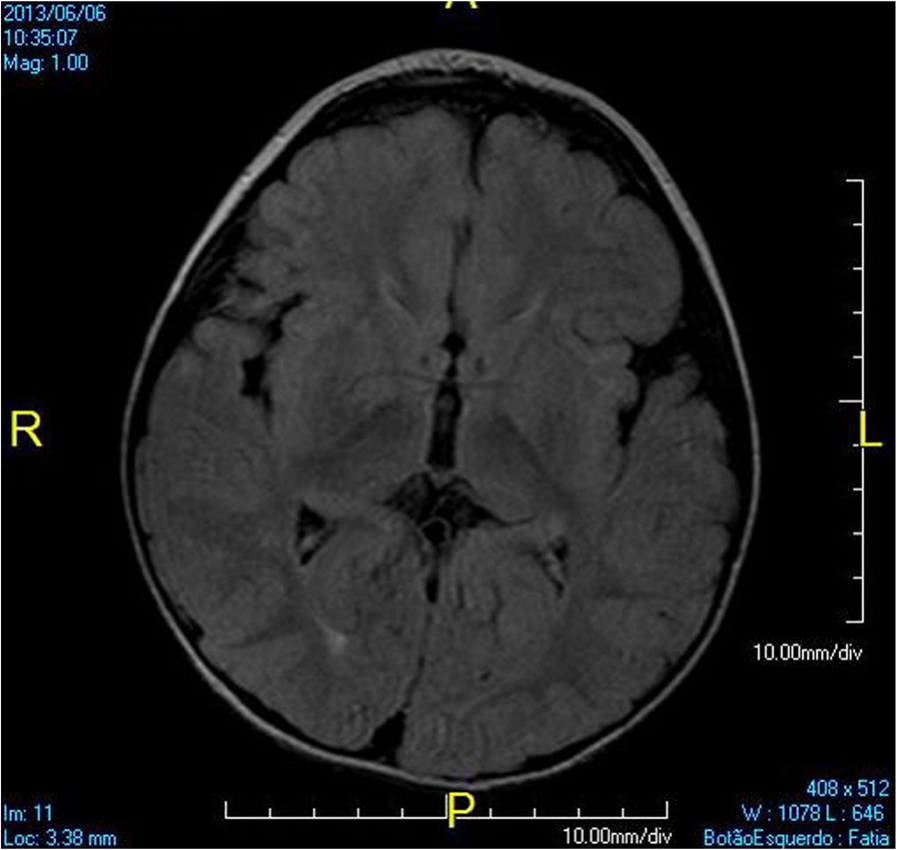
Brain MRI - Increased signal in FLAIR images in the withe matter territories adjacent to the lateral ventricles bodies and subcortical zone

**Fig. 6:**
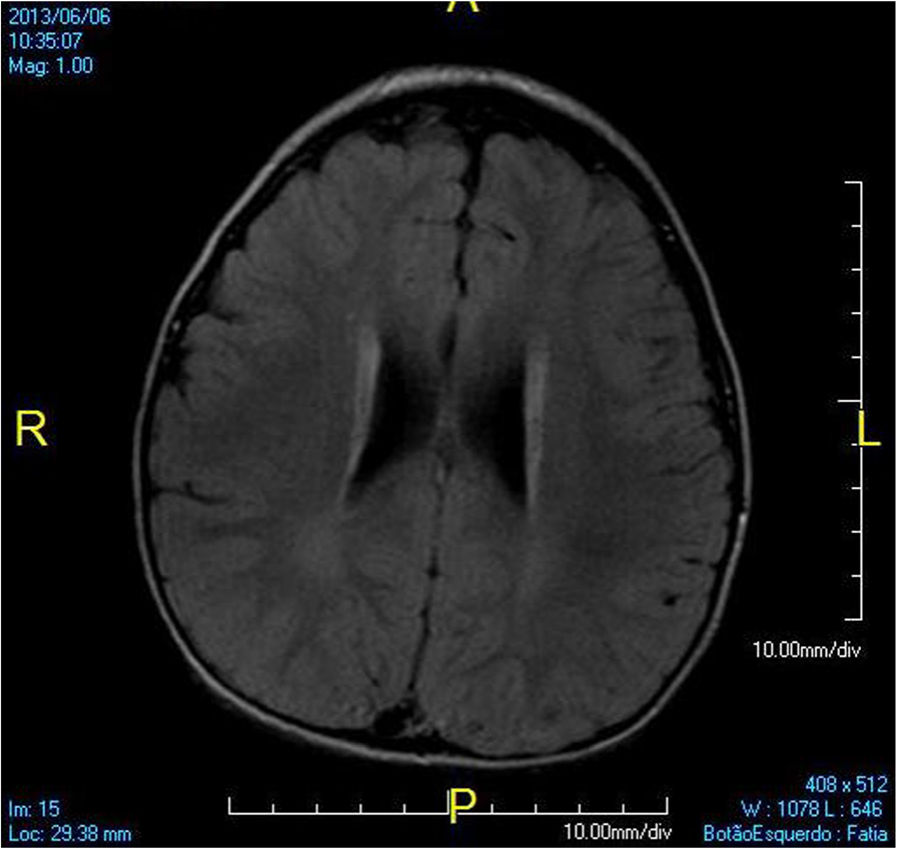
Brain MRI - Increased signal in FLAIR images in the withe matter territories adjacent to the lateral ventricles bodies and subcortical zone

**Fig. 7:**
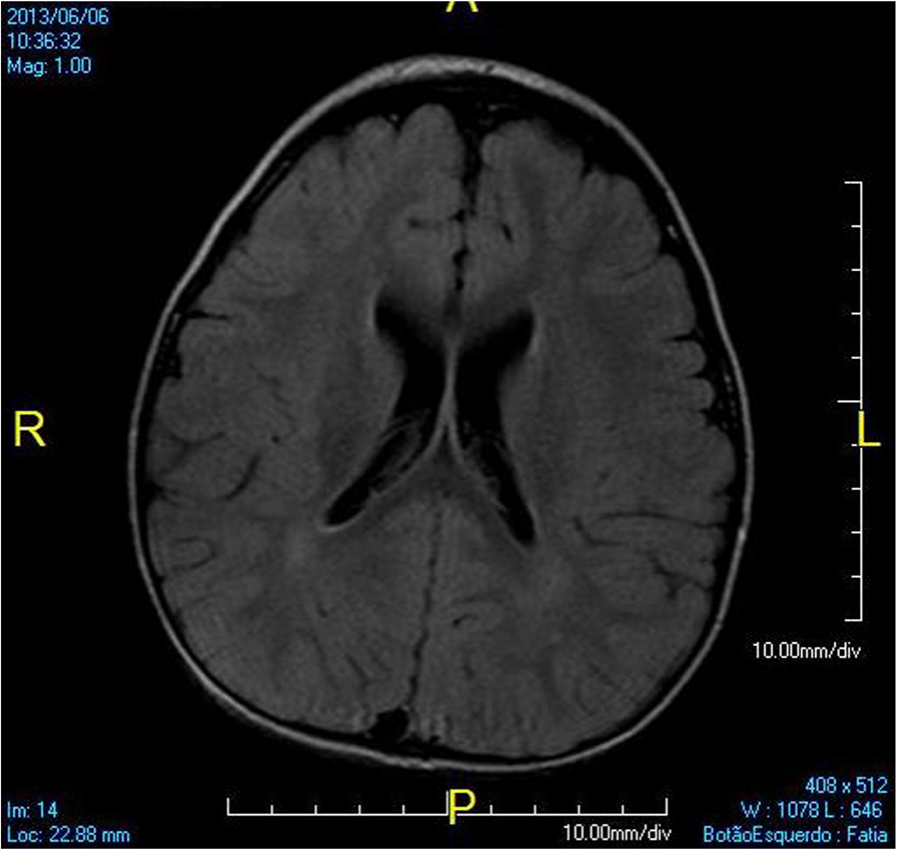
Brain MRI - Increased signal in FLAIR images in the withe matter territories adjacent to the lateral ventricles bodies and subcortical zone

**Fig. 8:**
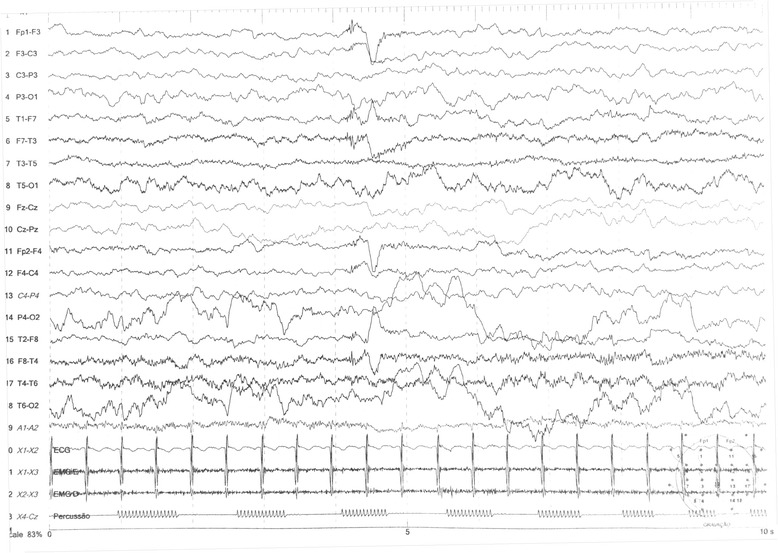
EEG showing non specific slow background activity, as well as no epileptiform discharges

At 3 years old, a brain MRI, which was undertaken to investigate the patient’s global developmental delays, showed no signs of abnormal patterns in myelination or in the setup of the structures comprising the supra and infratentorial brain compartments. However, we identified sparse increased signal in FLAIR and T2-weighted images in the white matter territories adjacent to the lateral ventricles bodies and subcortical zones (Figs. 4, 5, 6, 7). In addition, around the same period of time, a v-EEG demonstrated signs of a non-specific slow background, but no other abnormal electrographic activity had been identified (Fig. 8).

Fragile X DNA screening, as well as metabolic screening for Inborn Errors of Metabolism results, all came back negative. Chromosome analysis was carried out (please, see below).

In order to reinforce our thoughts on the diagnosis of ASD related to the genotype found in this patient, we revisited the ASD diagnosis at the patient’s age of 4 years, according to the Childhood Autism Rating Scale (CARS) [15]. The overall CARS score was 44, suggesting that the child was in the severe range of the autistic spectrum. This finding confirmed our primary developmental diagnosis of ASD, which has finally been aligned to the novel DSM5 criteria.

### Data analysis

A high resolution G-banded chromosome analysis of peripheral blood lymphocytes showed 46,XX,del(12p)(13.2) karyotype (550–600 GTW bands) in this patient.

The 12p13.2 deletion has not been identified in the parent’s karyotype, indicating a *de novo* terminal deletion in the short arm of chromosome 12.

We also used OMIM database to scrutinize scientific data related to all genes seated on the 12p13.2 region. Then, we selected the genes that could potentially be related to one of the etiologies of ASD.

### Review of the Literature

Aside from the present patient, we found an additional number of 43 cases described in the medical literature since 1974, that have been related to the 12p region. However, to the best of our knowledge, none of the previous had been specifically linked to the 12p13.2 band. All cases were summarized in Table 1.

**Table 1:** Behavioral and clinical phenotypes in the 12p interstitial and distal deletion spectrum syndromes

Cases	Deleted segment	ASD	ID	Other	Dysmorphic features
Mayeda et al. 1974 [38]	12.13-pter	NA	+	-	+
Magnelli/Therman 1975 [39]	12p12-pter	NA	+	-	+
Teconi et al. 1975 [40]	12p	NA	-	+PMD	+
Malpuech et al. 1975 [52]	12p11-p12.2	NA	-	+PMD	+
Orye & Craen 1975 (1) [41]	2p12	NA	+	+PMD	+
Orye & Craen 1975 (2) [41]	2p12	NA	-	+PMD	+
Magenis et al. 1981 [34]	12p12.3	NA	+	+PMD	+
B.-Dartigalongue et al. 1985 [42]	12p11-p12.1	NA	+	+PMD	+
Kivlin et al. 1985 [43]	12p12.2-pter	NA	-	-	+
Romain et al. 1987 [53]	12p13.1-13.3	NA	+	-	+
Baroncini et al. 1990 [44]	12p13-pter	NA	-	-	+
Fryns et al. 1990 [45]	12p11.2-12p13.1	NA	-	+PMD	+
Nagai et al. 1995 [54]	12p11.21-p12.2	NA	+	-	+
Bahring et al. 1997 [55]	12p11.21-p12.2	NA	-	-	+
Baker et al. 2002 (1) [46]	12p	NA	+	+NS	+
Baker et al. 2002 (2) [46]	12p	NA	-	+PMD/LD	NA
Glaser et al. 2003 [14]	12p12.1-p12.3	NA	-	+PMD	+
Stumm et al. 2007 (1) [47]	12p11.21-p13.2	NA	-	-	+
Stumm et al. 2007 (2) [47]	12p11.21-p13.2	NA	-	-	+
Velinov et al. 2008 [48]	12p13.3-pter	NA	+	+NS	-
Lu et al. 2009 [56]	12p11.21-12p12.2	NA	_	+PMD	+
Rooryck et al. 2009 [49]	12p13.33	NA	+	NA	+
McDonald et al. 2010 [20]	12p	NA	+	+	+
Abdelmoity et al. 2011 (1) [50]	12p13.33	-	-	+ADHD/ID/SE	+
Abdelmoity et al. 2011 (2) [50]	12p13.33	-	-	+ADHD/ID/SE	-
Abdelmoity et al. 2011 (3) [50]	12p13.33	-	-	+PMD/LD	NA
Talkowski et al. 2011 [58]	12p13.1	-	+	+Seizures	NA
Soysal et al. 2011 [12]	12p11.1-12.1	NA	+	-	+
De Ligt et al. 2012 [57]	12p13.1	-	+	+Language Delay	NA
Dimassi et al. 2012 (1) [35]	12p13.1	-	+	+LD/PMD/Seizures	+
Dimassi et al. 2012 (2) [35]	12p13.1	-	+	+Language Delay	+
Dimassi et al. 2012 (3) [35]	12p13.1	+	+	+PMD/Language Delay	+
Thevenon et al. 2012 (1) [36]	12p13.33	-	-	+PMD/Language Delay	+
Thevenon et al. 2012 (2) [36]	12p13.33	-	-	+Language Delay	NA
Thevenon et al. 2012 (3) [36]	12p13.33	+	+	+Language Delay/ADHD/LD	+
Thevenon et al. 2012 (4) [36]	12p13.33	-	-	+Language Delay/ADHD	-
Thevenon et al. 2012 (5) [36]	12p13.33	-	+	+ADHD/LD	-
Thevenon et al. 2012 (6) [36]	12p13.33	+	+	+Language Delay/ADHD/LD	+
Thevenon et al. 2012 (7) [36]	12p13.33	-	+	+PMD/Language Delay/ADHD/Anxiety	+
Thevenon et al. 2012 (8) [36]	12p13.33	-	+	+PMD/Language Delay/ADHD/LD/Anxiety	+
Thevenon et al. 2012 (9) [36]	12p13.33	-	+	+Language Delay/LD	+
Vargas et al. 2012 [11]	12p13.33	NA	ID	+PSY	+
Hoppe et al. 2014 [13]	12.2p11.22	NA	+	+PMD	+
Present Patient	12p13.2	+	+	+LD/PMD	-

+Feature present; − Feature negative; *LD* learning disability, *ADHD* attention deficit hyperactivity disorder, *ADD* attention deficit disorder, *ID* intellectual disability, *PMD* psychomotor delay, *Psychosis* PSY, *SE* staring episodes, *NS* non specific, *NA* not available

Ref. [11, 13, 20, 34–36, 38–40, 42–49, 51–58]

## Discussion

In this report, we described a new case of a young male child with initial global developmental delay which turned out to become more specific of the typical core signs that underpin the diagnosis of ASD, as the child became a preschooler. These signs are characterized by deficits in social and communication capabilities associated with repetitive behaviors and activities plus circumscribed interests [1].

According to recent analysis, it appears that there is a growing body of evidence pointing toward an increasing rate of ASD with a current average prevalence of 1 % worldwide [16]. The higher rates of ASD might be the by-product of a variety of factors, ranging from the heterogeneity in the diagnostic criteria and diagnostic practice, to changes in the epigenetic factors [3, 17, 18].

When it comes to the genetic influences on the etiology of ASD, one has to take into account the heterogeneity of genotypes, comprising roughly one thousand genes or so, that have been associated with autism. According to Butler et al. [19], routine cytogenetic studies typically identify abnormalities in chromosomes 2, 3, 4, 5, 7, 8, 11, 13, 15, 16, 17, 19, 22 and X. Those findings include deletions, duplications, translocations and inversions involving specific chromosome regions where known candidate ASD genes are seated [19]. Noteworthy, even considering the fact that this is an updated publication, the 12p deletion spectrum is still not mentioned as a common site for genes related to ASD. Moreover, according to McDonald et al. [20], aside from the gene-enriched subtelomeric regions in these most common sites, 1p, 22q, 4p, 9q, 8p, 2q and 20p, respectively outlined here in order of frequency, there have been only a few reports involving the short arm of the chromosome 12. Table 1 illustrates all patients with the interstitial and terminal 12p deletions previously described since the first publications in 1974.

In any case, our 4-year-old patient has a genetic setup in 12p region that, to best of our knowledge, has not yet distinctively been presented in the literature. That also includes the fact that this patient lacked the variable dysmorphic features frequently presented in the majority of related papers as listed in Table 2. In addition, although data on ASD was not available in many of the outlined cases in Table 1, aside from the present child, three of others displayed were described as having ASD. Noteworthy, this child is the only one of the four who did not display significant dysmorphic features.

**Table 2:** Frequent dysmorphic signs and associated congenital anomalies previously described in the 12p deletion syndrome

Head, face and neck	Thorax and abdomen	Genitals	Miscellaneous
Stenosis of the sagittal sutura Broad and webbed neck Facial asymmetry/oval shape Arched eyebrows Down slanted palpebral fissures Short Palpebral fissures Almond shape palpebral fissures Epicanthic folds Sclerocornea Eyelid coloboma Large, low set and hyperplastic ears, posteriorly rotated ears Microtia/anotia Large and flat nasal bridge Long philtrum Everted vermillion of the lowe lip Cleft lip and palate Hypoplastic mandible Micrognathia Broad chin Hypoplastic teeth and enamel Hyperplastic gengiva Protunding tongue	Asymmetric thorax Wide-set mamillae Hypoplastic lungs Atrial septal defect Low set umbilicus Multicystic dysplastic Kidneys Vertebral anomalies Extremities Short upper arms Cubitus valgus Short hands Brachymetaphalangy Clinodactyly/camptodactyly Squared firngertips Broad nails Broad thumbs Short metatarsal bones Big overlapping toes with hypoplastic nails Transverse creases	Cryptorchidism Hypoplasia of external genitalia Neurological Microcephaly Brachycephaly Optical nerve atrophy Sensorineural hearing loss Spasticity Presence of Babinsky sign Increased deep tendon reflexes Epilepsy Muscle atrophy	Short stature Low body weight Inguinal hernia Osteogenesis imperfecta Stillborn Turner like stigmata Decreased LDHB activity Sacro-coccygeal dimple

*LDHB* Lactic Dehydrogenase B

Ref. [14, 34, 38, 39, 41–44, 47, 48]

On the other hand, there are other previously published reports on sporadic ASD originated from an NMDA-related gene, named *GRIN2B*, that is located in 12p13.1. However, those cases were related to point mutations and translocations as opposed to deletions occasionally found in the 12p region [21–23].

At this point, one might also inquire about some of the risk factors for a brain injury this child had, such as, for instance, antenatal exposure to alcohol and hypoglycemia during the child’s initial hours of life. Indeed, according to a recent meta-analysis from Tsang et al., the alcohol exposure could partially be accounted for the appearance of atypical behavioral, social and cognitive difficulties [24]. Moreover, the fact that the child did not present with the FAS features does not rule out the broader, secondary diagnosis of FASD, which comprises FAS, pFAS, ARND and ARBD [25].

On the other hand, as there are no reliable biological markers today to rule in or out FASD, we can only say at this point that, taking into account the previously described literature on the relationship between ASD and the 12p deletion spectrum, the former might well be at least partially considered as the causative factor for this child’s ASD diagnosis. Furthermore, the patient’s brain MRI findings are non-specific and the increased signal in FLAIR and T2-weighted images might most likely be related to zones of terminal myelination, rather than a lesion caused by alcohol and/or the minor asymptomatic neonatal hypoglycemic episode.

We acknowledge there is a technical limitation in our report due to the lack of specific laboratory expertise and materials. We have not been able to pursue further investigations in this child using more sophisticated techniques, such as the aCGH arrays. This hampered our understanding in which genes were missing in the 12p13.2 of our affected patient.

Nevertheless, when looking up into the 12p13.2 region on the OMIM database, we were able to identify relevant genes related to *Homo sapiens* (human) species, as outlined in Table 3.

**Table 3:** Distribution of genes located in the 12p region

Gene	Protein	OMIM	Function
*TNFRSF1A*	TNF receptor superfamily member 1A	191190	Activates NF-kappaB, mediate apoptosis, and function as a regulator of inflammation. Germline mutations of the extracellular domains of this receptor were found to be associated with the autosomal dominant periodic fever syndrome.
*LRP6*	LDL receptor related protein 6	603507	Through its interaction with the Wnt/beta-catenin signaling cascade, this gene plays a role in the regulation of cell differentiation, proliferation, and migration and the development of many cancer types.
*CLEC7A*	C-type lectin domain family 7 member A	606264	Functions as a pattern-recognition receptor that recognizes a variety of beta-1,3-linked and beta-1,6-linked glucans from fungi and plants, and in this way plays a role in innate immune response.
*GABARAPL1*	GABA type A receptor associated protein like 1	607420	Interacts with a cohort of 67 proteins, with extensive binding partner overlap between family members, and frequent involvement of a conserved surface on ATG8 proteins known to interact with LC3-interacting regions in partner proteins.
*CLEC1B*	C-type lectin domain family 1 member B	606783	Expressed in myeloid cells and NK cells, which express, multiple calcium-dependent (C-type) lectin-like receptors, such as CD94 and NKG2D that interact with major histocompatibility complex class I molecules and either inhibit or activate cytotoxicity and cytokine secretion.
*STYK1*	serine/threonine/tyrosine kinase 1	61143	Receptor protein tyrosine kinases, like *STYK1*, play important roles in diverse cellular and developmental processes, such as cell proliferation, differentiation, and survival.
*PRB4*	proline rich protein BstNI subfamily 4	180990	Encodes a member of the heterogeneous family of basic, proline-rich, human salivary glycoproteins. The encoded preproprotein undergoes proteolytic processing to generate one or more mature peptides before secretion from the parotid glands.
*PRH2*	proline rich protein HaeIII subfamily 2	168790	Encodes a member of the heterogeneous family of proline-rich salivary glycoproteins. The encoded preproprotein undergoes proteolytic processing to generate one or more mature isoforms before secretion from the parotid and submandibular/sublingual glands. Certain alleles of this gene are associated with susceptibility to dental caries.
*CLEC12A*	C-type lectin domain family 12 member A	612088	This gene encodes a member of the C-type lectin/C-type lectin-like domain (CTL/CTLD) superfamily which share a common protein fold and have diverse functions, such as cell adhesion, cell-cell signaling, glycoprotein turnover, and roles in inflammation and immune response. The protein encoded by this gene is a negative regulator of granulocyte and monocyte function. This gene is closely linked to other CTL/CTLD superfamily members in the natural killer gene complex region on chromosome 12p13.
*PRB1*	proline rich protein BstNI subfamily 1	180989	Encodes a member of the heterogeneous family of basic, proline-rich, human salivary glycoproteins. This gene is located in a cluster of closely related salivary proline-rich proteins on chromosome 12.
*TAS2R43*	taste 2 receptor member 43	612668	Belongs to the large TAS2R receptor family. TAS2Rs are expressed on the surface of taste receptor cells and mediate the perception of bitterness through a G protein-coupled second messenger pathway.
*PRB2*	proline rich protein BstNI subfamily 2	168810	Encodes a member of the heterogeneous family of basic, proline-rich, human salivary glycoproteins. The encoded preproprotein undergoes proteolytic processing to generate one or more mature isoforms before secretion from the parotid glands.
*KLRA1P*	killer cell lectin like receptor A1, pseudogene	604274	This locus was originally considered to be protein coding, but has been reclassified as a transcribed pseudogene because all associated transcripts are candidates for nonsensemediated decay (NMD).
*TAS2R31*	taste 2 receptor member 31	612669	Belongs to the large TAS2R receptor family. TAS2Rs are expressed on the surface of taste receptor cells and mediate the perception of bitterness through a G protein-coupled second messenger pathway
*PRB3*	proline rich protein BstNI subfamily 3	168840	Encodes a member of the heterogeneous family of basic, proline-rich, human salivary glycoproteins. The protein isoforms encoded by this gene are recognized as the “first line of oral defense” against the detrimental effects of polyphenols in the diet and pathogen infections.
*PRH1*	proline rich protein HaeIII subfamily 1	168730	Encodes a member of the heterogeneous family of proline-rich salivary glycoproteins. The encoded preproprotein undergoes proteolytic processing to generate one or more mature isoforms before secretion from the parotid and submandibular/sublingual glands.
*CLEC9A*	C-type lectin domain family 9 member A	612252	*CLEC9A* is a group V C-type lectin-like receptor (CTLR) that functions as an activation receptor and is expressed on myeloid lineage cells.
*TAS2R50*	taste 2 receptor member 50	609627	Belongs to the large TAS2R receptor family. TAS2Rs are expressed on the surface of taste receptor cells and mediate the perception of bitterness through a G protein-coupled second messenger pathway.
*CLEC1A*	C-type lectin domain family 1 member A	606782	Encodes a member of the C-type lectin/C-type lectin-like domain (CTL/CTLD) superfamily. Members of this family share a common protein fold and have diverse functions, such as cell adhesion, cell-cell signaling, glycoprotein turnover, and roles in inflammation and immune response. The encoded protein may play a role in regulating dendritic cell function.
*TAS2R46*	taste 2 receptor member 46	612774	Belongs to the large TAS2R receptor family. TAS2Rs are expressed on the surface of taste receptor cells and mediate the perception of bitterness through a G protein-coupled second messenger pathway.
*TAS2R20*	taste 2 receptor member 20	613962	Encodes a member of the taste receptor two family of class C G-protein coupled receptors. Members of this family are expressed in a subset of taste receptor cells, where they function in bitter taste reception, as well as in non-gustatory cells including those of the brain, reproductive organs, respiratory system, and gastrointestinal system.
*MAGOHB*	mago homolog B, exon junction complex core componente	-------	Findings show 2 genes *MAGOH* and *MAGOHB* are expressed in mammals; in macrophages, expression of *MAGOHB* but not *MAGOH* mRNA increases after LPS stimulation; both *MAGOH* proteins interact with other exon junction complex (EJC) components, incorporate into mRNAbound EJCs and activate nonsense-mediated decay
*TAS2R19*	taste 2 receptor member 19	613961	No description
*TAS2R30*	taste 2 receptor member 30	613963	No description
*CLEC12B*	C-type lectin domain family 12 member B	-------	No description
*PCS*	Parotid proline-rich salivary protein Pc	168710	No description
*TMEM52B*	transmembrane protein 52B	-------	No description
*MANSC1*	MANSC domain containing 1	-------	No description
*TAS2R12P*	taste 2 receptor member 12 pseudogene	--------	No description
*SMIM10L1*	small integral membrane protein 10 like 1	--------	No description
*TAS2R18P*	taste 2 receptor member 18 pseudogene	--------	No description
*TAS2R63P*	taste 2 receptor member 63 pseudogene	--------	No description
*TAS2R64P*	taste 2 receptor member 64 pseudogene	--------	No description
*LOH12CR2*	loss of heterozygosity, 12, chromosomal region 2 (non-protein coding)	--------	No description
*TAS2R15P*	taste 2 receptor member 15 pseudogene	--------	No description
*TAS2R67P*	taste 2 receptor member 67 pseudogene	--------	No description
*PR@*	proline rich protein gene cluster	--------	No description
*MORF4L1P2*	mortality factor 4 like 1 pseudogene 2	--------	No description
*HSPE1P12*	heat shock protein family E (Hsp10) member 1 pseudogene 12	--------	No description
*RNU7-60P*	RNA, U7 small nuclear 60 pseudogene	--------	No description
*RPL21P100*	ribosomal protein L21 pseudogene 100	--------	No description
*LINC01252*	long intergenic non-protein coding RNA 1252	--------	No description
*SLC25A39P2*	SLC25A39 pseudogene 2	--------	No description
*RNU6-545P*	RNA, U6 small nuclear 545, pseudogene	--------	No description
*RN7SKP161*	RNA, 7SK small nuclear pseudogene 161	--------	No description
*IQSEC3P2*	IQ motif and Sec7 domain 3 pseudogene 2	--------	No description
*DDX55P1*	DEAD-box helicase 55 pseudogene 1	--------	No description
*HNRNPABP1*	heterogeneous nuclear ribonucleoprotein A/B pseudogene 1	--------	No description
*LOC255308*	eukaryotic translation initiation factor 2 subunit gamma pseudogene	--------	No description
*LOC102724020*	uncharacterized LOC102724020	--------	No description
*LOC101928100*	uncharacterized LOC101928100	--------	No description
*LOC440084*	hCG1655019	--------	No description
*LOC101928162*	uncharacterized LOC101928162	--------	No description
*LOC440082*	uncharacterized LOC440082	--------	No description

Ref. [9, 26–34]

If one takes into account the theoretical factors (synaptogenesis, synaptic connectivity, dendritic spines formation and maintenance, neuronal membrane protein turnover and related neurotransmitters metabolism, as well as immunological issues) related to the pathogenesis of ASD, from the genes directly linked to humans, as enlisted in Table 3, one finds it important to observe that *TNFRSF1A* [26]*, LRP6* [9, 27], *CLEC7A* [28], *GABARAPL1 *[29]*, CLEC1B* [30], *STYK1* [31]*, CLEC12A* [32], *CLEC1A* [33]*, MAGOHB* [34], could be potentially involved in the pathogenesis of ASD, due to their anti-inflammatory, immunologic and neuro trafficking roles. However, given our limitation to further assess these data, more extensive and in-depth research is needed in that regard.

Below we describe some of the already known variations of clinical presentation in the 12p deletion spectrum that might be related to ASD.

### The 12p11.1-p12.1 interstitial deletion

In the report of Soysal et al. [12], a very distinctive phenotype is observed in a 12 years old girl with a karyotype 46,XX, del(12)(p11.1-p12.1). This patient, born from a young unrelated couple with an unremarkable family history, presented with dysmorphic craniofacial features (microcephaly, ocular hypertelorism, down-slanting palpebral fissures, strabismus, myopia, minor inner epicantal folds, arched eyebrow, broad nasal base, bulbous nose, short philtrum, microretrognathia, irregular tooth alignment), corporeal dysmorphic features (distal phalangeal abnormalities, 5th finger camptodactily, brachydactyly of the feet, scolioses and joint hyper mobility), ID and ASD. The most important genes seated in that region are *PKP2, ALG10, KRAS, FGD4, PTHLH, DNM1L*. However, an additional 0.191 MB deletion in 2p16.3 was found using aCGH microarray. *NRXN1* is considered the most significant gene in this region. According to Soysal et al. [12], the role of neurexin genes in synaptogenic activities has been previously attributed as a cause of ASD as well as other developmental and psychiatry disorders. The social deficits and behavioral abnormalities in this patient could most likely be a combination of influences coming from both regions. Therefore, a clear understanding in the role of deletion 12p11.1-p12.1 on ASD and related developmental disorders is not fully doable in this case.

### The 12p13.1 interstitial deletion

Dimassi et al. [35] reported on 3 intellectually disable patients with 12p13.1 deletions. The molecular findings had been initially investigated with aCGH technique and posteriorly confirmed via FISH and qPCR tests. Overall, they consisted in deletion of exon 1 and exon 2 of *GRIN2B.* This gene encodes the NR2B subunit of NMDA receptors, known to play a role in corticogenesis, neuronal migration and synaptogenesis during brain development. According to Dimassi et al. [35], there have been a few other reports in patients with ASD and ID but no facial dysmorphic features (oval-shaped face, arched eyebrows, almond-shaped palpebral fissures, long philtrum, everted vermillion border of the lower lip and broad chin) were noticed. In their paper, according to the description of their 3 patients (Table 1), one was considered autistic and 3 intellectually disabled. Furthermore, it is noteworthy to mention that only one also presented with seizures.

### The 12p13.33 distal subtelomeric deletion

In 2010, McDonald et al. [20] described a 40 week term baby born from non-consanguineous parents with an unremarkable family history, and after an uneventful pregnancy. At the age of 6 years, microcephaly, short nose, prominent ears were detected. In addition, the child had cognitive and social difficulties. Despite having tested with a normal karyotype, an MLPA test was further carried on using SALSA P070 kit (MRC Holland). The results indicated a deletion in the subtelomeric region, which was further confirmed by FISH and aCGH tests. The later revealed a 2.95 MB deletion in the region comprised of 36 genes, 16 of which having clinical significance according to OMIM. Although a more detailed description of the patient’s social and behavioral difficulties is lacking in this report, it appears as though this child could potentially meet clinical criteria for the diagnosis of ASD, but this information is presumptive and not subject to a reliable confirmation at this time.

Recently, another group of acknowledged genetic researchers from Brazil published a report of an 8-year-old male patient, who was also born with spina bifida [8]. The child was diagnosed with ASD by age 2,5 years. At that time, the diagnosis was made on the basis of the former DSM-IV-TR criteria and had also been sustained by the CARS. This boy had a 1.5 Mb microdeletion in 12p13.33 zone, which encompasses 13 genes, one of them, the *ERC1*, a 500 kb gene known to molecularly regulate neuroplasticity and neurotransmitters in a presynaptic level [8, 36]. By comparing previous reports [36, 37] of patients and taking into account the implications of synaptic dynamics over the casual course of ASD, Silva et al. raised awareness to the role of *ERC1* as part of the growing body of genes that can potentially be accounted for the etiology of ASD [8].

## Conclusion

The 12p deletion spectrum is rarely described as part of the selective genotypes thought to be related to ASD. Even inside of a small piece of the puzzle, there might be ample variation in the behavioral and clinical phenotypes of children and adults presenting with this particular genetic profile.

In that regard, the particular 12p13.2 distal deletion presentation is one of the possible genotypes encompassed by the “12p deletion spectrum syndrome”, that might potentially be connected to the diagnosis of ASD and related developmental disorders.

Due to their role in the inflammatory, immunologic and neuro trafficking routes, the genes *TNFRSF1A, LRP6, GABARAPL1, CLEC1B, STYK1, CLEC12A, CLEC1A, MAGOHB*, and *CLEC7A,* that comprise part of 12p13.2 band, might potentially play a role in the pathogenesis of ASD. More extensive research is needed to clarify the later.

## Abbreviations

aCGH: Microarray-based comparative genomic hybridization
ARBD: Alcohol related brith defects
ARND: Alcohol related neurodevelopmental disorders
ASD: Autism spectrum disorders
CARS: Childhood autism rating scale
CNV: Copy number variation
FAS: Fetal alcohol syndrome
FASD: Fetal alcohol spectrum disorder
FISH: Fluorescence in Situ Hybridization
ID: Intellectual disability
MLPA: Multiplex-ligation dependent-probe-amplification
MRI: Magnetic resonance image
NMDA: N-Methyl-D-Aspartate
OMIM: Online mendelian inheritance in men
pFAS: Partial fetal alcohol syndrome
qPCR: Quantitative real time polymerase chain reaction
SNV: Single nucleotide variation
T2W: T2-weighted images
v-EEG: Videoelectroencephalogram
